# Fragile X-Associated Diminished Ovarian Reserve and Primary Ovarian Insufficiency from Molecular Mechanisms to Clinical Manifestations

**DOI:** 10.3389/fnmol.2017.00290

**Published:** 2017-09-12

**Authors:** Limor Man, Jovana Lekovich, Zev Rosenwaks, Jeannine Gerhardt

**Affiliations:** The Ronald O. Perelman and Claudia Cohen Center for Reproductive Medicine, Weill Cornell Medicine New York, NY, United States

**Keywords:** fragile X syndrome, fragile-X-associated primary ovarian insufficiency, diminished ovarian reserve, *FMR1* premutation carriers, fragile-X-associated diminished ovarian reserve

## Abstract

Fragile X syndrome (FXS), is caused by a loss-of-function mutation in the *FMR1* gene located on the X-chromosome, which leads to the most common cause of inherited intellectual disability in males and the leading single-gene defect associated with autism. A full mutation (FM) is represented by more than 200 CGG repeats within the *FMR1* gene, resulting in FXS. A FM is inherited from women carrying a FM or a premutation (PM; 55–200 CGG repeats) allele. PM is associated with phenotypes distinct from those associated with FM. Some manifestations of the PM are unique; fragile-X-associated tremor/ataxia syndrome (FXTAS), and fragile-X-associated primary ovarian insufficiency (FXPOI), while others tend to be non-specific such as intellectual disability. In addition, women carrying a PM may suffer from subfertility or infertility. There is a need to elucidate whether the impairment of ovarian function found in PM carriers arises during the primordial germ cell (PGC) development stage, or due to a rapidly diminishing oocyte pool throughout life or even both. Due to the possibility of expansion into a FM in the next generation, and other ramifications, carrying a PM can have an enormous impact on one’s life; therefore, preconception counseling for couples carrying the PM is of paramount importance. In this review, we will elaborate on the clinical manifestations in female PM carriers and propose the definition of fragile-X-associated diminished ovarian reserve (FXDOR), then we will review recent scientific findings regarding possible mechanisms leading to FXDOR and FXPOI. Lastly, we will discuss counseling, preventative measures and interventions available for women carrying a PM regarding different aspects of their reproductive life, fertility treatment, pregnancy, prenatal testing, contraception and fertility preservation options.

## Introduction

Fragile X syndrome (FXS) represents the most common cause of inherited intellectual disability in males. It is also the leading single-gene defect associated with autism. The fragile X mental retardation (*FMR1*) gene is located near the end of the long arm of the X chromosome, locus Xq27.3. It includes a CGG (Cytosine-Guanine-Guanine) trinucleotide repeat within the 5′ untranslated region. The name FXS arises from the characteristic chromosomal fragility in that locus observed during karyotyping (Sutherland and Ashforth, 1979).

A normal, unaffected gene contains less than 45 CGG repeats while having between 45 and 54 repeats is classified as intermediate, or gray zone, as this is when some level of CGG repeat instability in the gene transmission to the next generation has been reported (Nolin et al., 1996). The range of 55–200 CGG repeats is considered a premutation (PM) and more than 200 CGG repeats are categorized as a full mutation (FM), resulting in FXS (Kronquist et al., 2008). A recent meta-analysis reported the PM prevalence in the general population to be 1:150–300 females and 1:400–850 males (Hunter et al., 2014). Interestingly, the prevalence varies between different racial/ethnic groups; it is the highest in Colombia and Israel (1:100 females), and lowest in Japan (1:1674 females) (Seltzer et al., 2012; Hunter et al., 2014). The FXS prevalence is estimated to be 1 in 4000 males (Turner et al., 1996) and 1 in 8400 females (Pesso et al., 2000).

The PM is associated with increased level of *FMR1* gene transcription but decreased translation, resulting in low to normal levels of fragile X intellectual disability protein (FMRP) (Tassone et al., 2000b; Hagerman and Hagerman, 2002). The *FMR1* allele containing a FM is affected by DNA hypermethylation of the promoter and the CGG repeat region, causing its inactivation- transcriptional silencing. As a consequence, there is no FMRP production, which results in FXS in males (Fu et al., 1991; Heitz et al., 1991; Pieretti et al., 1991; Verkerk et al., 1991; Sutcliffe et al., 1992). In female FM carriers, however, due to random inactivation of one X chromosome, mRNA can be transcribed from the normal, but not the mutated-methylated allele, leading to lower but measurable FMRP levels.

In the past, individuals with fragile X mutations were divided either into affected (with more than 200 CGG repeats) or unaffected individuals (≤200 CGG repeats). Advances in diagnostic methods and increased awareness, however, have led to stratification of the previously “unaffected” group into normal, gray zone and PM, with associated clinical manifestations. This appears reasonable to consider the clinical spectrum of symptoms associated with fragile X as a continuum (McConkie-Rosell et al., 2005). The PM is associated with disorders distinct from FXS, including fragile-X-associated tremor/ataxia syndrome (FXTAS), an adult-onset neurological disorder (Hagerman et al., 2001) affecting primarily males, as well as fragile-X-associated primary ovarian insufficiency (FXPOI), and the fragile-X-associated diminished ovarian reserve (FXDOR) in female carriers: all of which we will expand upon in this review. In the recent years, more characteristic phenotypes associated with a PM have been recognized: attention deficit hyperactivity disorder (ADHD), autism spectrum disorders (Farzin et al., 2006), intellectual disability, childhood seizures (Bailey et al., 2008; Chonchaiya et al., 2012), adult-onset psychiatric conditions (Franke et al., 1996; Roberts et al., 2009), migraine headaches (Au et al., 2013), immune-mediated disorders, mainly thyroid disorders (Winarni et al., 2012), hypertension, fibromyalgia (Leehey et al., 2011) and chronic muscle pain (Rodriguez-Revenga et al., 2009).

The FXS phenotype, divergent from phenotypes associated with PM, varies by sex; males being more severely affected due to the X-linked inheritance and having only one X chromosome. Some of the distinctive facial features associated with FXS include an elongated face with a prominent forehead and large protruding ears, macrocephaly, strabismus, high arched palate with an occasional cleft palate. The facial characteristics often develop over time. Other symptoms include enlarged testicles (macroorchidism) and connective tissue disorders (hyper-flexible joints; hyperextensible fingers, thumbs and wrists). The cognitive phenotype is characterized by a spectrum of features including developmental delay, intellectual and learning disabilities. The behavioral phenotype includes ADHD, speech and language delay, anxiety and autism spectrum disorders (McConkie-Rosell et al., 2005). Affected females may have a subtle phenotype, which makes it sometimes hard to establish the diagnosis based on clinical features alone. Up to 50% of females with a FM have some characteristic physical features associated with FXS. The intellectual impairment is usually less severe than observed in affected male (McConkie-Rosell et al., 2005).

Another sexual disparity when it comes to FM is the male’s capability of reproducing. Hagerman et al. (2001) and Hagerman and Hagerman (2002) state that males with FXS have been documented to be fertile and capable of reproduction. On the other hand, Crawford states that most affected males do not reproduce, presumably due to the severity of intellectual disability (Crawford et al., 2001). This alleged disparity may be reconciled when we take the cognitive function into account. The majority of males with FXS are intellectual disable, with severity ranging from profound (IQ < 20) to mild intellectual disability (IQ 50–70), with most being moderately disabled (IQ 40–54) (Hagerman and Hagerman, 2002). Male affected with the FM can reproduce, this is usually seen in about 15% of the FXS males, who have an IQ at 70 or higher. On the other hand, females are often less affected, most probably because of random X-inactivation, and are therefore at risk of transmitting a FM to their progeny. Interestingly, women with a PM have an increased risk of FXPOI compared to that of FM patients (Allingham-Hawkins et al., 1999; Uzielli et al., 1999). In this review article, we will elaborate on: (i) the clinical manifestations of POI, in specific the ovarian dysfunction found in female PM carriers; (ii) propose the definition of FXDOR (distinct from FXPOI); (iii) review recent scientific findings that might shed light on some potential mechanisms leading to FXDOR and FXPOI; and (iv) discuss counseling, preventative measures and interventions available for women carrying a PM regarding different aspects of their reproductive life, fertility treatment, pregnancy, prenatal testing, contraception and fertility preservation options.

## Clinical Manifestations of POI in General and Ovarian Dysfunction in PM Carriers in Particular

### POI- Primary Ovarian Insufficiency, and FXPOI

Normal ovarian function is a result of a continuous process that commences with primordial germ cells (PGC) formation, proliferation and migration, through the development of follicular units during fetal life (Baker, 1963). It then extends into neonatal and adult life, characterized by a steady follicular loss or atresia, and ends with a physiologic insufficiency of the ovary, or menopause (Faddy et al., 1992; De Felici et al., 2005). The ovary is susceptible to various intrinsic and extrinsic factors that might impair its normal formation and/or function i.e., genetic defects, smoking (Cooper et al., 1995), environment and medical issues including endometriosis, chemotherapy, radiotherapy, or ovarian surgery (De Vos et al., 2010; American College of Obstetricians and Gynecologists Committee on Gynecologic Practice and Practice Committee, 2014; Rossetti et al., 2017; Vabre et al., 2017). The extreme form of ovarian dysfunction is manifested as POI.

POI is a diagnosis that accentuates the extreme spectrum of an impaired ovarian function. The term POI was coined by Albright et al. (1942), when he reported on a cluster of symptoms including amenorrhea, estrogen deficiency and menopausal follicle stimulating hormone (FSH) levels in 11 young women. He used this term to emphasize that the primary defect in this cohort was within the ovary. Today we still use his basic understanding for diagnostic purposes with some modifications. First, serum estrogen is not a mandatory criterion for the diagnosis. Many, but not all, women with POI develop symptoms of estrogen deficiency, including hot flashes, vaginal dryness and sleep disturbances. For example, lack of symptoms of estrogen deficiency might be due to intermittent ovarian function. On the other hand, some women experience hot flashes despite continued regular menses. Second, amenorrhea no longer represents the only criterion to characterize disturbance of menstrual cyclicity. With a better understanding of the phenotypic extent of POI, inclusion criteria have broadened and now include any cycle irregularities (oligomenorrhea, polymenorrhea, menometrorrhagia, dysfunctional uterine bleeding and amenorrhea- primary/secondary) which persist for more than four consecutive months. Nowadays, POI is characterized by the triad of cycle irregularities, as elaborated above, for at least 4 months, and two recordings of elevated levels of FSH >40 IU/L at least 1 month apart, in a woman younger than 40 (Coulam et al., 1986; Welt, 2008). POI can be primary, spontaneous, or secondary to external insults.

Although quite often used synonymously, POI should not be equated with menopause. The main difference lies in the fact that with POI, ovarian function can still be present albeit unpredictable and/or intermittent. Moreover, it is believed that roughly 50% of women with POI retain intermittent ovarian function for many years, may exhibit spontaneous follicular development, and commence menstruation (Rebar and Connolly, 1990). This is strongly supported by the fact that 5%–10% of women with POI can conceive (van Kasteren and Schoemaker, 1999) and deliver a child (Rebar et al., 1982; Nelson et al., 2005) without any medical intervention, even years after diagnosis was established. Similarly, Hipp et al. (2016) reported that 12.6% of women diagnosed with FXPOI conceived spontaneously after diagnosis. The time to conception after diagnosis ranging up to 12 years (Hipp et al., 2016). Approximately 20% of women with *FMR1* PM will develop FXPOI. Among women with idiopathic sporadic or the rare form of familial POI, about 2%–6%, and 14%, respectively, carry the PM within the *FMR1* gene (Sherman, 2000; Sullivan et al., 2011). It’s unknown whether the risk of FXPOI is higher in women carrying the PM who also have a family history of POI in comparison to those who don’t.

POI is a multi-factorial disease, which affects about 1% of women under the age of 40 (Coulam et al., 1986). For the most part (90%), the cause cannot be determined (idiopathic), whereas approximately 10% have an etiology that can be identified. *FMR1* PM is one of the most common single-gene mutation causes of POI in women with a normal karyotype. POI associated with the *FMR1* gene PM is referred to as FXPOI. Other known single-gene mutations associated with POI are Bone morphogenetic protein 15 (BMP-15), Diaphanous homolog 2 (DIAPH2) and Inhibin alpha subunit (INHA).

Most probably, POI occurs through two main mechanisms: (i) inadequate formation of the follicular pool *in utero*; and (ii) abnormally extensive or fast depletion of the follicular pool via atresia during post-natal (neonatal, childhood and adult) life. Thus, it would be logical to conclude that POI would be preceded by some degree of DOR. The term “POI” can be described as a continuum of compromised ovarian function over time, rather than a dichotomous state (normal ovarian function followed by an early menopause). Ovarian function is to deteriorate over a period of months to years and progress from an occult stage, which may manifest only by reduced fecundity, through a phase of biochemical manifestation (also elevated FSH levels), reaching the final stage of overt ovarian insufficiency characterized by irregular or absent menses, along with reduced fecundity and elevated FSH levels (Welt, 2008).

An important consideration when diagnosing POI is that this diagnosis is usually devastating and life-changing for many women (Greil, 1997; Nelson, 2009). Indeed, impaired self-esteem, shyness, social anxiety and low level of social support are more common in women facing POI (Schmidt et al., 2006; Orshan et al., 2009). Taking into consideration that PM carriers have an increased risk of depression and anxiety, it was recommended by Nelson et al. (2005) that these women return for follow-up to screen for symptoms of depression and anxiety and in general be encouraged to find sources of emotional support.

### Ovarian Dysfunction in PM Carriers

There is no apparent difference in age at menarche between PM carriers and healthy controls (Allen et al., 2007). Even so, the reproductive span is reduced in the former. First clinical hints for impaired ovarian function might be found during adolescence when approximately 3% of the PM carriers will experience non-specific menstrual cycle irregularities (De Caro et al., 2008). Schwartz et al. (1994) noted for the first time that PM carriers reported irregular menses more often than non-carriers. The fact that PM carriers have shorter menstrual cycles in comparison to age-matched women additionally supports the idea that FXDOR precedes FXPOI (Table 1, Welt et al., 2004). Hormonal profile alterations might be evident as well.

**Table 1:** Menstrual cycle and hormonal milieu characteristics of premutation (PM) carriers compared to age-matched regularly cycling women.

	PM carries (*n* = 11)	Controls (*n* = 22)	*P* value
Total cycle length in days	26.1 ± 1.0	28.2 ± 0.4	*P* < 0.05
Follicular phase length in days	12.9 ± 0.8	14.5 ± 0.4	*P* < 0.05
Luteal phase length in days	13.2 ± 0.5	13.7 ± 0.3	NS
Mean follicular FSH levels IU/L	21.9 ± 3.5	11.2 ± 0.5	*P* < 0.001
Luteal FSH levels IU/L	14.6 ± 3.9	7.9 ± 0.5	*P* < 0.001
Follicular Inhibin B levels pg/ml	77 ± 11	104 ± 6	*P* < 0.05
Luteal Inhibin B levels pg/ml	35 ± 5	41 ± 3	*P* < 0.05
Follicular Inhibin A levels IU/ml	1.6 ± 0.2	2.1 ± 0.2	*P* < 0.05
Luteal Inhibin A levels IU/ml	3.4 ± 0.7	5.8 ± 0.5	*P* < 0.05

*Adapted from Welt et al. (2004). Summarizes the cycle and follicular phase duration in days, in addition to the mean follicular and luteal levels of follicle stimulating hormone (FSH), Inhibin A and Inhibin B. Study group, comprising regularly cycling fragile X PM carriers, 24–41 year old (34.5 ± 5.7 year), was compared with age-matched, regularly cycling controls, 23–41 year old (34.6 ± 5.8 year), at each stage of the cycle. All values are presented as mean ± SEM*.

FSH is a pituitary hormone, which stimulates the growth and recruitment of immature ovarian follicles. With ovarian aging and diminishing number of follicles, less Inhibin is being released from the ovary, which consequently weakens FSH negative feedback resulting in increased release of FSH. An elevated level of FSH on the 3rd day of menstrual cycle, therefore, indicates diminution in the ovarian pool and has been used as a marker of ovarian reserve (aging) for decades (Scott et al., 1989). PM carriers demonstrate significantly higher FSH levels in the follicular phase (cycle day 1–10) when compared to healthy, age-matched women (Murray et al., 1999). Considerably higher serum FSH levels were also found in the follicular and luteal phases in PM carries. Furthermore, lower Inhibin A and Inhibin B levels have also been discovered in these patients, implying impaired follicular and luteal ovarian function (Table 1, Welt et al., 2004). Anti-Mullerian hormone (AMH), another marker of ovarian reserve, was measured by Rohr et al. (2008) and it appeared to be more sensitive than FSH in identifying an early decline in ovarian function among PM carriers. A subtle decrease in AMH levels in women carrying the PM was detected as early as the age of 18 years, in the absence of differences in FSH levels between controls and carriers, suggesting a low ovarian reserve for PM carriers even at this young age (Rohr et al., 2008). These findings support the notion of a continuous deterioration of ovarian function in these patients that may be detected only by sensitive biochemical markers.

There is an increased rate of infertility in women carriers compared to non-carriers (Allen et al., 2007). Despite the early DOR, no increase in the rate of miscarriages or chromosomal abnormalities due to maternal age-related chromosomal nondisjunction was demonstrated in offspring of women carrying a PM (Murray et al., 2000a; Allen et al., 2007). Thus, while there may be a relative drop in follicle number, oocyte competence continues to be related to chronological age. Lastly, these women enter menopause, on average, 5 years earlier than the women in general population (Partington et al., 1996; Murray et al., 2000a; Sullivan et al., 2005). Because of these substantial impairments associated with carrying a PM, the American College of Medical Genetics (ACMG) recommends testing for the *FMR1* PM in all women with POI (Wittenberger et al., 2007). We would make the case for testing all women presenting with any reproductive dysfunction (Zev Rosenwaks, personal communication).

### Correlation between Number of Repeats and Ovarian Function

Some evidence suggests a correlation between CGG repeat length and severity of the phenotype. CGG repeat length had previously been associated with FXTAS clinical features, such as the age of onset of tremor and executive dysfunction (Cornish et al., 2011). The repeat size is also considered a risk factor for developing FXTAS dementia (Seritan et al., 2016). Others reported a correlation between age and CGG repeat length, as they found that male carriers with over 100 CGG repeats are more susceptible to the effects of aging on measures of executive function (Cornish et al., 2011). Furthermore, similar correlations were found in other repeat expansion diseases. For instance, in Huntington’s disease, longer CAG repeat length is associated with earlier onset of the disease (Ross and Tabrizi, 2011).

Interestingly, in female PM carriers, there appears to be a difference in the degree of ovarian function among different CGG repeat length subgroups. Although having below 45 repeats is considered normal, some studies have shown a direct correlation between the number of repeats and DOR (Bretherick et al., 2005; Bodega et al., 2006; Gleicher et al., 2009). However, other studies have refuted this finding (Schufreider et al., 2015; Pastore et al., 2017). Notably, the official statement of the ACMG is that a repeat length lower than 45 is not associated with an abnormal phenotype (Monaghan et al., 2013).

This is not the case when it comes to the PM CGG repeat range (55–200). Women carrying a PM exhibit impaired fertility compared to that observed in non-carriers (Allen et al., 2007). Women with a mid-sized PM (approximately 80–100 repeats) are at greater risk of developing FXPOI (Ennis et al., 2006; Allen et al., 2007; Wittenberger et al., 2007). It appears that the risk increases with increasing PM repeat size between 59 and 99, while it actually declines with >100 repeats (Sullivan et al., 2005). Additionally, when it comes to *in vitro* fertilization (IVF) treatment, it has been shown that fewer eggs are retrieved from PM carriers when compared those of age-matched controls carrying less than 55 CGG repeats (Elizur et al., 2014). In agreement with Sullivan’s findings, Bibi et al. (2010) reported that PM carriers with less than 100 repeats demonstrate a lower response to controlled ovarian hyperstimulation (COH) and decreased fertilization rate, in comparison to those with more than 100 CGG repeats.

## Proposing A New Definition; FXDOR- Fragile X-Associated Diminished Ovarian Reserve

The concept of ovarian reserve defines the women’s reproductive potential as a function of a number and quality of her remaining oocytes (Practice Committee of the American Society for Reproductive Medicine, 2015). DOR is a condition in which the ovary loses its normal reproductive potential, compromising fertility. The condition may result from disease or injury, but most commonly occurs as a result of normal aging. Overt POI might take several years to develop unless it’s secondary to removal of the ovaries, chemotherapy or radiotherapy. On the other hand, DOR is not an overt phenotype and harder to diagnose because of its subtle nature, thus, as of today, there is no consensus on the definition of DOR (Ferraretti et al., 2011). However, compared with women of similar age, women with DOR commonly have regular menses but a reduced quantity of ovarian follicles. Therefore, patients with DOR may have a limited response to ovarian stimulation with fertility medications and reduced fecundity (Committee on Gynecologic Practice, 2015). Also, evidence of DOR does not necessarily equate with the inability to conceive (Practice Committee of the American Society for Reproductive Medicine, 2015).

We would like to propose a new term for PM carriers with reduced ovarian reserve: “FXDOR”. We believe this term is more clinically appropriate, as it best corresponds to this process of continuous deterioration along with its fluctuant nature. The diminished ovarian function/reserve, might or might not lead to overt FXPOI (depending on whether amenorrhea occurs at the age of 40, or later). FXDOR encompasses the phases of ovarian insufficiency previously termed by Welt as “biochemical” and “occult” (Welt, 2008). This diagnosis will be a diagnosis of exclusion, after excluding all other known reasons for infertility (for instance, male factor, endometriosis, mechanical factor, etc.), in a woman carrying a PM allele, with regular menstruations regardless to the levels of ovarian markers, younger than 40 years of age will be considered to suffer from FXDOR. There is no established gonadotrophin concentration cutoff to suggest the initiation of ovarian insufficiency (Panay and Kalu, 2009), most probably due to the fluctuant and reversible nature of the ovarian function. The difference between these two stages, of the occult and biochemical, is FSH levels, which might fluctuate. Therefore, it seems reasonable to utilize a single unifying term-FXDOR. Also, there is no difference in the clinical management of both stages either, so the division to these two categories becomes redundant and cumbersome.

As of now, the prevalence of FXDOR remains undetermined, as the clinical presentation is subtle and often associated with no symptoms besides possible subfertility or infertility, it might be completely asymptomatic (unlike FXPOI, characterized by alarming menstrual irregularities). If a PM carrier has already completed childbearing at a much younger age before developing significant FXDOR affecting fertility, or if she never attempted conceiving (lack of interest, delaying childbearing for socio-economic reasons), FXDOR might progress completely unnoticed, and the first symptom of her PM might present as FXPOI. Any other phenotypic features associated with a PM are non-specific as well, and wouldn’t be alarming enough to justify genetic testing. Given that the definition of PM and having intermediate alleles are related to the potential for generational expansion and not of the possible ovarian function, the only way to reach a comprehensive understanding of the scope and determining especially the cutoff of the repeat size would be to screen the general population for FXDOR.

## Recent Scientific Findings Proposing Possible Mechanisms for Ovarian Dysfunction in PM Carriers

Successful development of primordial follicles during fetal life is critical for the establishment of the ovarian reserve, which in itself determines woman’s reproductive lifespan. In order to detect the link between the genetic impairment and the phenotype, we need to have a deeper understanding of the mechanisms involved. The etiology of ovarian dysfunction in carriers of the PM remains elusive. Elucidating mechanisms responsible for the development of FXDOR and FXPOI have proven to be challenging, largely due to the scarcity of suitable human samples and the lack of appropriate animal models available for research. Although FXDOR precedes FXPOI, it seems that only a portion of women having the phenotypic expression of FXDOR will eventually exhibit the extreme form of FXPOI. Expanded CGG repeats in the PM range are linked to the occurrence of both FXDOR and FXPOI; hence it is highly likely that the same mechanism accounts for both. Nevertheless, the alternative explanation that different mechanisms are involved in the development of FXDOR and FXPOI cannot be completely ruled out at this time.

Limitations and restrictions on the availability of human ovarian tissue and therefore existing studies on the mechanism leading to ovarian dysfunction in PM women force us to extrapolate findings from research in the field of FXTAS (Bourgeois et al., 2011; Seritan et al., 2013) onto FXDOR and FXPOI. One of the most commonly observed features in brain tissue of FXTAS patients are the ubiquitin-positive intranuclear inclusions (Galloway and Nelson, 2009). These inclusions are composed of proteins and RNA. The presence of the FMR1 mRNA in the intranuclear inclusions (Tassone et al., 2004) together with the observed increase of FMR1 mRNA in PM carriers (Tassone et al., 2000a) led to the suggestion that a toxic RNA gain-of-function mechanism might be responsible for the development of FXTAS. It was proposed that the mutant FMR1 mRNA containing the expanded CGG repeats might sequester several RNA-binding proteins, preventing them from performing their normal intracellular functions (Galloway and Nelson, 2009). Willemsen et al. (2003) described an increase in both the number and the size of the inclusions during the course of life, which correlates with the progressive character of the cerebellar tremor/ataxia syndrome in humans. This suggests a correlation between the presence of intranuclear inclusions in distinct regions of the brain and the clinical features in symptomatic PM carriers (Willemsen et al., 2003).

A similar mechanism was suggested previously as a cause of two other repeat expansion disorders: myotonic dystrophy type 1 (DM1) and type 2 (DM2) (Mirkin, 2007). For DM1, it has been demonstrated that the RNA-binding protein MBLN co-localizes with the *DMPK* gene transcript containing the expanded repeats (Fardaei et al., 2001). This caused dysregulated splicing of MBLN targets and DM1 phenotype in transgenic mice (Kanadia et al., 2003). In addition, evidence of diminished ovarian reserve was reported in women with DM1 (Srebnik et al., 2014). It is possible that an accumulative process of RNA-protein complex over time represents the basis of this late-onset FXPOI as well. This observation suggests that a similar accumulation could perhaps occur in the ovary and may correlate with the onset and severity of the phenotype.

Besides the discovery of RNA aggregates in cells of FXTAS patients, ubiquitin-positive inclusions containing an FMR polyglycine protein (FMRpolyG) were found in brain cells of these patients (Todd et al., 2013). These intranuclear neuronal inclusions are generated by repeat-associated non-UTG (RAN)-initiated translation. RAN translation was also reported to occur in other repeat expansion diseases such as spinocerebellar ataxia type 8, ALS and frontotemporal patients (C9ALS/FTD) (DeJesus-Hernandez et al., 2011; Ash et al., 2013; Cleary and Ranum, 2014). Similar to the toxic RNA aggregates, FMRpolyG could sequester specific viable factors for proper cell function through protein-protein interaction. Furthermore, it was suggested that impairment in the protein quality control pathway, which is necessary for the cells in order to get rid of toxic and misfolded proteins, could contribute to the CGG repeat associated toxicity in human cells (Oh et al., 2015). In addition, reducing translation of FMRP was observed in PM carriers, probably due to decreased translation efficiency of the mutant FMR1 mRNA (Tassone et al., 2000a). FMRP is an RNA-binding protein which shuttles between the nuclear and cytoplasmic compartments (Jin and Warren, 2000). There is evidence that FMRP acts as a translational suppressor and functions in a dose-dependent manner as a regulator of gene expression at the post-transcriptional level (Laggerbauer et al., 2001; Li et al., 2001). Reduced FMRP might cause some of the symptoms in PM women, such as a reduced germ cell population as seen in *Drosophila* model system.

**Figure 1:**
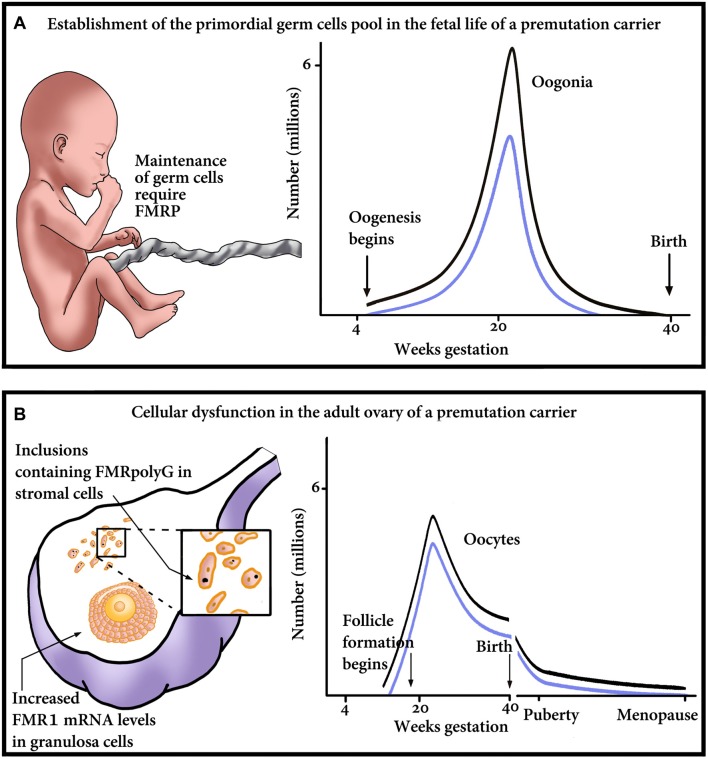
Proposed model of major points of gonadal impairment consequent to *FMR1* premutation (PM) presence during fetal and adult life. **(A)** Establishment of the primordial germ cell (PGC) pool in a fetus with a PM. Due to reduced FMRP, the final endowment, as well as maintenance of the PGC, could be affected. The graph outlines the relationship between the number of oogonia and gestational age till birth. Damage to the PGCs and consequent reduction in the PGC pool size will result in reduced number of oogonia, thus shifting the graph downwards, from the non-carrier range (black line) to the PM carrier range (blue line). **(B)** Cellular dysfunction in the adult ovary of a PM carrier. The ovary suffers damage at the cellular level, which is clinically manifested as a diminished ovarian reserve. As seen on the left, the impairment occurs in the granulosa as well as the stroma cells of the ovary. Reduction in the number of oocytes/follicles could occur as a consequence of the mRNA-induced granulosa cell toxicity and subsequent dysfunction, detrimental effect of the inclusions containing FMRpolyG on the stroma cells, or both. On the right, a schematic representation of the number of oocytes, in non-carriers (black line) or PM patients (blue line). The graph emphasizes that the PM carriers’ ovaries contain fewer oocytes than non-carriers’, at any age.

The question remains whether a decrease in the number of primordial follicles arises from an insult during germ cell development or is it a result of an increased velocity of a diminishing oocyte pool by atresia or follicular destruction. Correspondingly, which mechanistic explanation is compatible with the phenotype of FXDOR and FXPOI? Is it a formation of abnormal potentially gonadotoxic RNA or protein aggregates during oocyte development, or later, during post-natal life? It is also possible that the explanation lies in a failure of the follicle to respond to gonadotropin stimulation. Herein we are about to propose some possible mechanisms that might explain the phenotype of FXDOR and FXPOI. The mechanisms postulate optional damage *in utero*, at the level of the establishment of the PGC, or later in postnatal life and the adult ovary (Figure 1).

### Maintenance of PGC Require FMRP

During embryonic development at 6–8 weeks, germ cells begin to divide rapidly. By 16–20 weeks, fetal ovaries contain 6–7 million follicles, reaching its peak. In a mouse model, it was established that FMRP is expressed in PGC in the fetus (Hergersberg et al., 1995). It was also found that the PM allele does not affect the establishment of the primordial follicles pool (Sherman et al., 2014). This advocates that the expanded CGG repeats do not interfere with the assembly and the creation of the follicles. However, Yang et al. (2007) using *Drosophila* as a model, found that FMRP is required for preservation of germline stem cells (GSCs) in the ovaries. Ovaries of *Drosophila* female are composed of ovarioles. Each ovariole contains a functional unit called a germarium and differentiated egg chambers. GSCs are located at the tip of the germarium, and along with normal development, divide asymmetrically. GSCs generate some daughter cells for self-renewal, while other GSCs are displaced from the niche and become cystoblasts, which bud off the germarium as individual egg chambers and sustain oogenesis (Spradling et al., 1997). *Drosophila* ovaries of the *FMR1* null mutant contained fewer egg chambers and in some insistences, the germaria were completely empty. These results indicate that in *Drosophila* FMRP is required for the maintenance of GSCs (Yang et al., 2007).

In concord with this observation, it has been shown in fetal ovarian samples that human FMRP is expressed in germ cells surrounded by FMRP-negative pregranulosa and interstitial cells (Rifé et al., 2004). FRMP expression in these germ cells coincides with the loss of expression of the pluripotency-associated protein (Rosario et al., 2016). Although the function of FMRP in fetal ovaries is unknown, a reduction of FMRP in the PM germ cells could affect the volume of the follicular pool. It has been shown that the translation of FMRP was strongly inhibited in cells containing the PM. This inverse correlation between decreased FMRP with increased repeat length is probably due to reduced translation efficiency of the mutant FMR1 mRNA (Primerano et al., 2002). The reduction in efficient translation thought to be caused by secondary mRNA (hairpin loops) structures disrupts proper ribosomal scanning, causing stalling at the expanded CGG repeat of *FMR1* (Fu et al., 1991; Tassone et al., 2000a). This reduction of FMRP expression could influence germ and stem cell maintenance, and lead to a reduced follicular pool in PM patients. Thus far, these models were not tested in humans.

Nonetheless, an international collaboration (Allingham-Hawkins et al., 1999) has established that in contrast to PM women, FXS patients have no elevated risk for FXPOI in comparison to that of the general population. However, it is uncertain whether FXS patients are at risk of developing FXDOR. FMRP, even though decreased, is still present in PGC of FM patients since the *FMR1* locus is unmethylated in fetal oocytes (Malter et al., 1997). FMRP could play a role in the maintenance of the PGCs during fetal development and establishment of the follicle pool (Figure 1A). Reduction of FMRP in PM and FM individuals might conclude in less PGCs and a decreased follicle pool to begin with. Nevertheless, the rate of attrition could be normal, therefore, no FXPOI phenotype is apparent in FXS patients in contrast to PM carriers. PM, in addition, to a reduction in FMRP have an increase in FMR1 mRNA, which could aggravate the ovarian dysfunction even further. Alternatively, there could be disparities between species and FMRP may not play a substantial role in the determination of the size of the follicular pool and consequently ovarian function in humans: therefore, FMRP could be less detrimental than the presence of toxic mRNA found in PM patients.

### Increased mRNA Levels in Granulosa Cells of PM Women

During normal folliculogenesis, FMRP is predominantly expressed in granulosa cells (Hinds et al., 1993; Hergersberg et al., 1995; Schuettler et al., 2011). Although using mouse models, it was found that the PM allele does not hinder the establishment of the primordial pool, the number of more advanced subclasses of follicles was reduced (Sherman et al., 2014). This observation suggests that expanded CGG repeats do in fact interfere with the follicle development and assembly of the follicular unit. A toxic effect in human granulosa cells was exhibited, when granulosa cells were transduced with mRNA containing CGG repeats in the PM range (Hubayter et al., 2009). Recent findings also reported increased FMR1 mRNA levels in mice ovary and human granulosa cells of PM carriers (Elizur et al., 2014; Sherman et al., 2014). Although the findings in PM women to date only show a correlation between the increased mRNA levels and low ovarian reserve, these results support a proposed toxic RNA-gain function mechanism similar to FXTAS in PM granulosa cells.

The mutant FMR1 mRNA could sequester proteins by the formation of secondary RNA structures. RNAs containing CGG repeats are known to adopt secondary structures such as intramolecular hairpins (Zumwalt et al., 2007). Proteins could bind to these non-canonical RNA structures forming RNA-protein aggregates in the granulosa cells. Loss of function of these RNA-binding proteins in cells could compromise cell integrity and lead to early follicular decay (Figure 1). It has been shown in FXTAS-affected cells that CGG repeats bind to a large number of proteins, including hnRNP A2, Pur**α**, Lamina A/C and the miRNA biogenesis complex Drosha/DGCR (Jin et al., 2007; Sofola et al., 2007; Sellier et al., 2013). Another example is the RNA-binding protein Sam68, which is recruited to the RNA aggregates, generated by the FMR1 mRNA containing the expanded CGG repeats. The sequestration of SAM68 causes an altered SAM68-regulated splicing in FXTAS patients (Sellier et al., 2010). Interestingly, SAM68 has been suggested to regulate the splicing of the mRNA of the FSH and the luteinizing hormone receptors (Bianchi et al., 2010). Indeed, altered splicing of these proteins could lead to ovarian resistance to FSH and LH at the receptor level.

In addition, an increased amount of FMR1 mRNA in granulosa cells could also lead to a rise in R-loop formation, a secondary DNA-RNA hybrid structure formed by the repeats. R-loops could trigger genome instability and induce early decay of the follicles in PM women. These structures were observed by using the recently developed R-loop antibody in PM cells and also FXS cells, in which the *FMR1* gene transcription was reactivated by treatment with the DNA methylation inhibitor 5-aza-29-deoxycytidine (Groh et al., 2014; Loomis et al., 2014). Increased R-loops formation could lead to an increase in DNA damage in the cells. The formation of R-loops results in exposure to an unpaired single-stranded DNA due to the RNA-DNA hybridization (Santos-Pereira and Aguilera, 2015). Single-stranded DNA is more unstable and susceptible to lesions and transcription-associated mutagenesis or transcription-associated recombination (Aguilera, 2002). Another potential consequence of R-loop formation is the induction of genomic instability by interfering with DNA replication (Gan et al., 2011; Castellano-Pozo et al., 2012). Collisions of the transcription machinery with the replication fork have been shown to induce DNA breaks in budding yeast and mammals (Prado and Aguilera, 2005; Gottipati et al., 2008; Boubakri et al., 2010). Furthermore, replication fork stalling was detected at expanded CGG repeat sites in FXS stem cells and at expanded GAA repeats in Friedreich’s ataxia stem cells, another repeat expansion disorder (Gerhardt et al., 2014, 2016). Prolong replication fork stalling could induce DNA breaks if unrepaired could result in cell apoptosis (Nowsheen and Yang, 2012). Yet, as of today, R-loops were not detected in human granulosa cells.

### Inclusions Containing FMRpolyG in Stromal Cells

Intranuclear inclusions seem to be common in neurodegenerative conditions. Chang et al. (2011) demonstrated ubiquitin-positive inclusions within nuclei of the ovarian stromal cells. These inclusions appear to represent the ovarian counterparts of similar structures seen in the neurons of FXTAS patients. Interestingly, it was described by Sellier et al. (2017) that FMRpolyG interacts with the nuclear lamina protein LAP2b and disorganizes the nuclear lamina architecture in neurons differentiated from FXTAS iPSCs. Recently, FMRpolyG in ubiquitin-positive inclusions were found in ovarian stromal cells of a PM women (Buijsen et al., 2016; Figure 1B). Hypothetically, protein aggregates could be responsible for ovarian dysfunction leading to FXDOR and FXPOI. Perhaps an abnormal function of the stromal cells in the ovary will cause follicular atresia and an early decay of the ovarian pool.

Surprisingly, no inclusions containing FMRpolyG were found in the follicles *per se* (Buijsen et al., 2016). Since increased FMR1 mRNA levels were observed in granulosa cells and FMRP seems to be expressed in all stages of the ovarian follicular development, we would expect a finding like this, even so, that wasn’t described. One explanation could be that follicles containing inclusion are damaged, become atretic and are cleared away. However, FMRpolyG inclusions were only studied in the ovaries of one single PM woman so far, these results should be confirmed in additional ovarian samples obtained from women carrying the PM.

### Correlation between Repeat Size and the Severity of the Phenotype

Sherman et al. (2014) describe a non-linear association between the number of CGG repeats and symptoms in FXPOI patients. However, the mechanism leading to this phenomenon is not clear. An increase of FMR1 mRNA level with the PM repeat length was observed in FXTAS-affected cells (Tassone et al., 2007) explaining the rise of symptoms with the repeat size until approximately 100 repeats. The only minimal decrease in the ovarian dysfunction in women with PM allele over 100 repeats could be explained by a different mechanism, skewing of the X-chromosome inactivation (García-Alegría et al., 2007). García-Alegría et al. (2007) found that the relationship between mRNA levels and repeat size is nonlinear; a significant positive correlation between CGG repeats and total mRNA levels has been found in the PM range <100 CGG, but this correlation diminishes from 100 onward. Nonetheless, when corrected for the X-inactivation ratio, García-Alegría et al. (2007) observed the mRNA levels increase as the number of CGG repeats increases, and this increase is highly significant over 100 CGG. They suggest that due to skewed X-inactivation, mRNA levels tend to normalize in females when the number of CGG repeats increases.

## Counseling, Preventative Measures and Interventions Available for Women Carrying A PM

### The Risk of Allele Expansion into a FM in the Next Generation and Clinical Implications

The PM can expand and be transmitted to the offspring in the form of a PM with a greater number of repeats or expand into a FM range. The pattern of inheritance suggests that the FM evolves during an intergenerational, multistep process characterized as anticipation (Pembrey et al., 1985). Both, the PM as well as the FM within the *FMR1* gene are inherited in an X-linked dominant fashion; female carriers transmit it to 50% of their offspring, while males transmit the PM to all of their daughters and none of their sons. The transmission to the offspring and its phenotype will depend on, the sex of the parent transmitting the gene, the sex of the child, the number of CGG repeats within the parental *FMR1* gene, and the stability of the affected allele, which depends on the presence of AGG trinucleotide interruptions within the allele.

A female PM carrier can transmit PM allele to both her male and female progeny. Due to its instability, the PM allele may expand into a PM with a higher number of repeats, or reach the range of a FM in the next generation (Nolin et al., 2011). The number of CGG repeats within the maternal premutated allele may undergo expansion during oogenesis (Malter et al., 1997) as well as during postzygotic mitoses in the embryo (Wöhrle et al., 1993). It has been long accepted that only maternally inherited PM can expand into a FM in the next generation. On the other hand, PM as well as FM fathers can transmit only a PM to their daughters (Fisch et al., 1995). It is also believed that a paternally inherited PM does not expand to the same extent as the one inherited from the mother and can frequently contract. In fact, almost 40% of daughters of male PM carriers have PM with a lower number of CGG repeats than their fathers, in comparison to only 2% of daughters whose PM are shorter than their carrier mothers’. Moreover, when the transmission from the father expands it’s by relatively fewer repeats compared to transmission from the mother (Fisch et al., 1995). A possible explanation lies in the fact that sequences with a high number of CGG repeats are highly unstable in the developing sperm and jeopardize their survival, as evidenced by only PM-size alleles found in spermatozoa of PM, as well as FM males (Reyniers et al., 1993). Nonetheless, this axiom has been challenged by at least one case report of a mentally disabled female child who inherited both a PM as well as a FM from her mosaic father (Zeesman et al., 2004). Although the father’s peripheral blood cells demonstrated mosaicism, both premutated and fully mutated *FMR1* allele were present, his spermatozoa only contained the premutated allele, suggesting that the expansion to a FM found in the girl must have occurred post-zygotically.

The phenotype of a female child with a FM, as a result of either inheriting a maternal PM that expanded or inheriting an actual FM, is variable, ranging from severe intellectual impairment to apparently normal functioning. A male child, on the other hand, will almost invariably exhibit features of the FXS unless the *FMR1* allele is hypomethylated like in “high functioning males”. When it comes to PM inheritance, female offspring are at risk of developing FXDOR or FXPOI regardless of the sex of the parent transmitting the PM (Murray et al., 2000b; Sullivan et al., 2005).

It has been previously demonstrated that the number of CGG repeats within the maternal PM allele is in direct correlation with the probability of expanding into a FM in the offspring (Fisch et al., 1995; Nolin et al., 2011). For instance, a maternal allele containing 55–59 CGG repeats carries a 3.7% risk of expanding into a FM in the next generation, as opposed to 98% if the allele contains ≥100 repeats (Nolin et al., 2011). The lowest number of CGG repeats reported to be associated with a single generation expansion into a FM was from a woman carrying a PM allele of 56 repeats (Fernandez-Carvajal et al., 2009). Women with an intermediate number of CGG repeats (45–54) do not transmit a FM, although expansion to a PM length in their offspring has been described (Nolin et al., 2011).

It has been shown that the number of AGG interruptions within the CGG repeat region is inversely correlated with the instability of a PM allele and the risk of its expansion to a FM (Eichler et al., 1994). Yrigollen et al. (2012) reported that the presence of AGG interruptions reduces the risk of transmission of a FM, specifically for maternal alleles containing <100 repeats. These findings were further validated by Nolin et al. (2015), strengthening the association between the number of AGG repeats with CGG repeat region stability and providing more accurate risk assessments of expansion to FM in the next generation for women with 45–90 CGG repeats within the *FMR1* allele. Nolin et al. (2015) found that in each CGG repeat size category, those without any AGG interruptions had the greatest risk of instability and expansion into a FM. For instance, if a female carrier, whose allele contains 55–59 CGG repeats, has at least one AGG interruption within the allele, the risk of expansion into a FM in the next generation is reduced from 3.7% to less than 1%.

### Preconception Counseling for Women Carrying the PM

Identifying PM in a timely fashion is of paramount importance. By doing so, two major problems associated with this disorder could potentially be avoided: (i) the development of FXDOR or FXPOI before childbearing, which could otherwise render conception difficult or even impossible; and (ii) the presence of a FM, and its clinical manifestations, in the offspring.

### Screening and Patient Counseling

The American College of Obstetricians and Gynecologists (ACOG) recommends PM carrier screening for women with a family history of fragile X-related disorders or intellectual disability, who are considering pregnancy or are currently pregnant (Committee on Genetics, 2017). The College also stresses the importance of testing women who present with unexplained ovarian insufficiency and/or menopausal-range FSH levels before the age of 40. Southern blot and polymerase chain reaction (PCR) are the preferred methods of determining the number of CGG repeats within the *FMR1* gene for either screening or diagnostic purposes.

In the same committee opinion, the ACOG stated that “conditions included in an expanded carrier screening panel should meet the following criteria: have a carrier frequency of one in 100 or greater, have a well-defined phenotype, have a detrimental effect on quality of life, cause cognitive or physical impairment, require surgical or medical intervention, and have an onset early in life” (Committee on Genetics, 2017). Although the prevalence of the PM is not greater than 1:100, and the phenotype of the PM carriers is not well defined, FXS phenotype is defined. The FXS and the molecular biology of the *FMR1* gene are significantly more complex than the other single-gene screening targets. In particular, the carrier state being screened for, the PM allele, is also disease causing, unlike the heterozygous carrier mutations screened for autosomal recessive diseases such as Cystic fibrosis (Grody, 2011). The course of the disease, as well as transmission to the next generation, can be influenced by medical intervention. Population-based carrier screening has been already implemented in certain countries that experience a higher incidence of this disorder (Geva et al., 2000). Just recently, Haque et al. (2016) reported on 346,790 individuals undergoing expanded carrier screening and provided insights on carrier frequencies for many rare conditions in a large, diverse, albeit selected population. The findings indicated that an expanded testing panel identified more hypothetical fetuses at risk for severe or profound phenotypes than did testing based on current screening guidelines. Moreover, this study brings additional data to the debate on population screening for FXS (Grody, 2011; Finucane et al., 2012). Interestingly, they reported that in every race/ethnicity category other than the Southeast Asian, FXS has been shown to be more common than spinal muscular atrophy, and more common than cystic fibrosis in all race categories. The authors suggest a reconsideration of FXS population screening (Haque et al., 2016). Given recent publications, and physician’s chance to intervene and improve the outcomes for these women on one side, and a relatively high incidence of this disorder in the general population on the other (Musci and Caughey, 2005; Berkenstadt et al., 2007), we support *FMR1* CGG repeat screening for all women of reproductive age.

Patients must have a clear understanding of what their results mean in order to be able to make informed decisions about their reproductive health or to prepare to care for an affected child. They should receive education and care tailored to their carrier screening results. Information regarding the likelihood of CGG repeat expansion, possibly to the level of a FM and its clinical consequences in subsequent generations should be discussed. In order to bypass the genetic inheritance risk, some couples may consider child-free living, no further children, adoption or foster care. Others may choose to use preventive measures; an egg/embryo or sperm donation from unaffected donors, or IVF with preimplantation genetic diagnosis (PGD) for the selection of unaffected embryos and a subsequent transfer. Of course, the couple can always decide to carry on with natural conception and perform fetal genetic testing or parent a child with FXS.

### The Risk of Development of FXDOR and Progression into FXPOI

As previously outlined, a relatively high proportion (up to 20%) of females carrying the PM will develop FXPOI (Sherman, 2000). Symptoms including menstrual irregularities or difficulty conceiving will not necessarily precede the cessation of ovarian function and the first presenting symptom may be secondary (or less commonly primary) amenorrhea. Even though spontaneous conception is possible in all POI patients (Rebar et al., 1982; van Kasteren and Schoemaker, 1999; Nelson et al., 2005), including FXPOI (Hipp et al., 2016), the overall chances of pregnancy are low, this devastating diagnosis represents one of the greatest challenges patients and reproductive endocrinologists face. Even though the majority of PM carriers will fortunately not develop FXPOI, they are at risk of acquiring FXDOR (Nolin et al., 2003, 2011). Regardless of the etiology, the vast majority of patients with a DOR will exhibit regular menstrual cyclicity, and the diagnosis is usually established during an infertility evaluation (Friese et al., 2006). Physiologic ovarian senescence, as well as the development of FXDOR and FXPOI, cannot be prevented or delayed. At this time, there is no known remedy that prevents continuous follicular atresia. In lieu of an overall increase of mean maternal age in the US as a result of delaying childbearing for socio-economic reasons (Mathews and Hamilton, 2016), identifying PM carriers early, stressing the importance of early childbearing, if possible is desired. Also, counseling them about possible consequences of delaying childbearing and fertility preservation options is of an essence. Women with a PM may feel pressured to pursue childbearing earlier than they planned due to the significant ramifications of the carrier state. Identifying the PM earlier would give these women the opportunity to make an informed decision regarding their reproductive and family planning.

### Genetic Counseling

All the individuals identified with either intermediate results or with CGG repeats in the PM or a FM range should be offered further genetic counseling (Committee on Genetics, 2017). During genetic consultation of a PM subject, the possible impact on other family members (female as well as male) should be emphasized. The counseling should explain the pathophysiology of the condition and educate a patient on possible clinical manifestations pertinent to her (such as cognitive impairment, FXDOR and/or FXPOI, FXTAS) as well as her future offspring (the possibility of expansion into a FM in the next generation and the risk of intellectual disability and autism). In the case of a PM, counseling should also encompass calculation of the risk of allele expansion into a FM range in the next generation, using the number of CGG repeats as well as the number of AGG interruptions within the maternal allele. Prenatal testing (PGD of embryos, or Chorionic villus sampling (CVS) or amniocentesis of a fetus) should be discussed and offered to any affected individual. Educating patients on the risk of FXDOR and FXPOI with associated infertility or subfertility is of paramount importance, as it might affect carriers’ family planning. Patients should be advised to consider earlier childbearing if feasible, or otherwise offered fertility preservation via oocyte and/or embryo cryopreservation. Women carrying the PM should be advised to avoid risk factors that are known to decrease the age at menopause, such as smoking. It should also be recognized that use of hormonal contraception may mask POI symptoms.

### Choosing the Right Diagnostic Test

Therapeutic and remedial options will depend on several factors: (i) age at diagnosis of a PM or FM of the affected individual; (ii) the risk of expansion to a FM in the following generation based on the number of CGG repeats and further refined by the number of AGG interruptions; (iii) ovarian reserve; and (iv) patient’s preference. Ideally, diagnosis of PM or a FM in the affected female should be established prior to conception, nevertheless, that is not always the case. It is not uncommon that the diagnosis is made during early pregnancy.

### Preimplantation Genetic Diagnosis

Establishing the diagnosis prior to conception, allows the performance of embryonic genetic testing and selection hence, avoiding the transfer of an embryo with abnormal CGG repeat, assuring that the offspring will have CGG in the normal range. Thus, virtually eliminating the possible need for termination of an otherwise affected pregnancy. PGD represents a technique by which embryos created via COH, oocyte retrieval and fertilized mainly by performing intracytoplasmic sperm injection (ICSI), are genetically tested and selected for embryo transfer based on the presence of the mutation of interest. The biopsy is performed on 1–2 blastomeres in the case of a 3-day embryo (Martin and Arici, 2008), or more (5–8) cells in the case of an embryo at the blastocyst stage, followed by a chromosomal or genetic analysis. The aim is to achieve a pregnancy with an unaffected embryo. Given the limited amount of genetic material obtained via this technique (6 pg of genomic DNA/cell), determination of the actual number of CGG repeats within the embryonic *FMR1* allele using single cell PCR can be associated with amplification failure (Malcov et al., 2007; Reches et al., 2009) and inability to accurately distinguish between the PM and the FM. Instead, the approach called linkage analysis is more commonly utilized. Linkage analysis relies on the principle that certain DNA sequences that are close together on a chromosome are less likely to be separated during chromosomal crossover, and are therefore inherited together. It requires genetic testing of the couple’s relatives (siblings, parents, or any living children) using either short tandem repeat (SRT) or less commonly single nucleotide polymorphisms (SNP) analysis, allowing an indirect identification of the affected maternal *FMR1* gene in the oocyte.

Even though it is costly, the major advantage of PGD is avoiding the need for termination of affected pregnancies. On the other side, one of the difficulties lies in the fact that a certain proportion of affected women exhibit FXDOR or FXPOI, which makes them less responsive to COH and can significantly diminish the availability of embryos for PGD. Fortunately, when PGD and embryo transfer were possible, the outcomes were comparable to those of other monogenic diseases (Tsafrir et al., 2010). Additionally, as the accuracy of PGD is 98%–99% (Liebaers et al., 2010), confirmation with prenatal testing (such as amniocentesis) later in pregnancy is recommended. Although determining the number of AGG interruptions within the affected allele might further stratify and lower the risk, it does not completely eliminate the risk of having a child with a FM. Additionally, PGD might reveal that all tested embryos are affected and are therefore not suitable for transfer. In this case, there are some other options available, which will be elaborated upon later in this manuscript.

### Prenatal Diagnosis

If either the risk of allele expansion into a FM is reasonably low (<5%) based on the number of CGG repeats and AGG interruptions; the PM or a FM diagnosis is established post-conceptionally; or the patient chooses not to proceed with PGD for any reason (prohibitively low ovarian reserve, the cost of treatment or simply patient’s preference), prenatal testing can be performed by fetal tissue sampling in the 1st or 2nd trimester. Its purpose is to identify a pregnancy with an affected fetus for termination or to prepare the parents for the birth of an affected child.

CVS is an invasive procedure by which placental cells are obtained for further genetic analysis. It is typically performed between 10–13 weeks of gestation, under ultrasound guidance. Depending on the location of the placenta, CVS can be performed either trans-abdominally or transvaginally. Its general risk of a miscarriage is <1% (Mujezinovic and Alfirevic, 2007). One of the advantages of CVS over amniocentesis is an earlier diagnosis, which allows for earlier termination of pregnancy when the procedure is less traumatic and generally associated with fewer complications. Even though the placenta and the fetus have the same embryonic origin and should, therefore, be genetically identical, this rarely might not be the case and they could contain genetically different cells lines. This phenomenon is called placental mosaicism. Performing genetic testing on placental cells, therefore, might not be an accurate representation of the genetic material of the fetus, and this can be avoided by amniocentesis that yields actual fetal cells.

Amniocentesis is an invasive procedure by which a small amount of amniotic fluid containing fetal cells is obtained for further genetic testing. It is typically performed between 15–20 weeks of gestation, under ultrasound guidance. It overcomes the previously mentioned issue of placental mosaicism and is associated with even lower risk of a miscarriage (Mujezinovic and Alfirevic, 2007). One of the disadvantages of amniocentesis is that it establishes the diagnosis in a later stage of pregnancy when termination is procedurally more difficult, generally associated with more complications, and requires a skillful operator.

Further laboratory testing is performed in the same manner, regardless of the source of the cells, either CVS or amniocentesis. The first step in the genetic analysis is the determination of the number of CGG repeats within the allele by PCR and categorizing it as either a normal, intermediate, PM or a FM. Southern blot is then used to more accurately distinguish a large PM from a FM and to determine the allele’s transcriptional activity by determining the extent of methylation. Alternatively, the CGG repeat length, AGG interruptions, and DNA methylation can be determined by AmplideX^®^ PCR (Asuragen). Until approximately 10 weeks of gestation, FMRP is expressed normally in FM males, whereas at 12.5 weeks it’s completely absent. FMRP expression in FM female >13 weeks is completely absent in a number of villi, whereas other villi show normal FMRP expression due to random X chromosome inactivation in females. X chromosome inactivation occurs very early in development before the villi start to proliferate, and it represents a clonal process. In addition, evidence indicates that X-inactivation occurs before the time of *FMR1* allele inactivation in the FM (Willemsen et al., 2002).

When analyzing cells obtained via CVS, the prenatal detection of the repeat number is accurate and reliable, but one should keep in mind that the methylation pattern observed in placental tissue retrieved at 10–12 weeks’ gestation is incomplete and might not reflect that observed in the live born (Iida et al., 1994). Occasionally, a follow-up confirmation with amniocentesis is required, as the test is accurate and reliable regarding both the methylation status, as well as the number of the repeats (McConkie-Rosell et al., 2005). Nevertheless, since PCR assays are so accurate and able to identify all FM, the consultation is based mainly on the repeat number. In addition, if the CVS is not conclusive, for instance, due to placental mosaicism, the possibility of a follow-up amniocentesis to clarify the status of the fetus should be discussed. One of the challenges of prenatal testing is the difficulty in predicting intellectual, psychological and behavioral phenotype in a female FM carrier, even when the methylation status is known, due to mosaicism for X-chromosome inactivation.

### Contraception

Women with FXDOR/FXPOI who do not wish to conceive should use contraceptives. As was published by Hipp et al. (2016), 12.6% of women diagnosed with FXPOI conceived spontaneously after diagnosis. Amazingly, the interval of time to conception after diagnosis was up to 12 years. It appears that there might be a temporary remission, which in rare cases may last for years. According to this data, we believe it is prudent to offer these patients appropriate contraception.

### Fertility Preservation Options

Although today, we can’t prevent or reverse the deterioration in the ovarian reserve, we do have a substantial experience in banking oocytes and embryos for the purposes of fertility preservation. That is the reason we believe that if a woman is diagnosed carrying a PM, she should be consulted regarding her risk of developing FXDOR and FXPOI, and be advised about her fertility preservation options. Two main options available for adult patients are oocyte or embryo banking.

## Conclusion

This manuscript encompasses recent scientific findings which have led to a better comprehension of the effect the *FMR1* PM on fertility. Lack of a deeper understanding of the *FMR1* PM mechanisms involved is holding us back in terms of treating and curing PM women and helping them restore or prevent further damage to their ovarian reserve. By continuously gathering evidence derived from animal and human models, we are always on our way to solving this puzzle. Using evidence supporting the importance of FMRP during embryonic life for maintenance of PGCs, and subsequently the involvement of both RNA and protein in the pathologic processes, we created a hypothesis, which could explain the chain of events leading to the reduction in ovarian reserve. We hypothesize that the phenotype is derived from the combination of damage occurring at different stages of development and maintenance of follicular pool: (i) at the level of PGCs establishment and formation during embryonic life; and (ii) post-natal damage occurring at the level of the ovary in the granulosa and stroma cells (follicle unit) (Figure 1). Nonetheless, environmental exposure, genetic background and lifestyle decisions will contribute to the phenotype as well. We propose that the severity of the ovarian damage is a reflection of the accumulation of multiple hits along the development and maintenance of the ovary throughout the course of life, from the embryonic stage until menopause.

The mechanisms leading to both FXDOR and FXPOI are probably, at least in part, the same. Even so, our understanding is only partial. The evidence support that the PGCs need FRMP for their maintenance, hence reduction of FMRP expression could influence the germ cells and stem cell maintenance, and lead to a reduction in the follicular pool in PM patients. Moreover, an early decay of the follicles could be a result of increased FMR1 mRNA or FMRpolyG protein toxicity through the sequestration of RNA- and non-RNA-binding proteins by the expanded CGG repeat length, thus leading to a functional insufficiency of the sequestered proteins. Another possible insult might be an increase in R-loop formation at the *FMR1* gene locus that results in DNA damage and cell death. Interestingly, the *FMR1* gene containing a PM remains unmethylated and the gene is transcribed, while FXS women have lower levels of FMRP expression. Despite the lower levels, FXS women do not suffer from ovarian dysfunction. These findings accentuate the fact that the role of FMRP in folliculogenesis is uncertain and needs to be elucidated.

Women carrying the PM have variable expression and face many challenges in their life, including menstrual abnormalities, infertility, the risk for bearing a child with a PM or a FM, and earlier menopause. Strikingly, these women are at risk for other conditions including dementia, hypothyroidism, hypertension, seizures, fibromyalgia, autoimmune disease, neuropathies, migraines and psychiatric conditions including postpartum depression. Even so, the magnitude of long-term risks associated with the disorder (including cardiovascular disease and osteoporosis) and the optimal means of reducing these risks are uncertain.

Undoubtedly, more research is needed on strategies to improve fertility outcomes for women carrying a PM. In the meantime, we see a benefit in determining the PM or the FM status earlier rather than later through a population-based screening program, as both of these conditions are actionable. Early detection will provide time for patient counseling and might affect individual’s decision making in order to prevent ovarian failure before childbearing has occurred. It also allows for prevention of having a child with FXS. Given the high incidence of both a PM and a FM in the general population, we strongly believe that this is the time to take a step forward and offer to screen all reproductive age women. It will be beneficial for the carriers to be informed, to understand the condition and ramifications, and to plan reproduction and/or fertility preservation accordingly.

## Author Contributions

JG, LM, JL wrote the manuscript and JG, LM, JL and ZR edited the article.

## Conflict of Interest Statement

The authors declare that the research was conducted in the absence of any commercial or financial relationships that could be construed as a potential conflict of interest.
